# Supplementary material for: Tolerability of 2 and 4 mg/kg Dosing Every 12 Hour of a Cannabidiol- and Cannabidiolic Acid-Rich Hemp Extract on Mixed-Breed Dogs Utilized for Teaching in a Closed Colony
Source: Animals (Basel). 2024 Jun 24;14(13):1863. doi: 10.3390/ani14131863 (PMC11240743; doi:10.3390/ani14131863)
Supplement: Supplementary file 1 [file animals-14-01863-s001.zip › animals-3048896-supplementary_updated.pdf]

Supplemental Figure S1. Student Survey for adverse events and behavioral changes figure of questions asked

Dog Name: ____ Treatment arm: ____________________

Student: ____ Date: ______________________________________

This assessment is related to a clinical assessment of a commonly use nutraceutical and is a placebo blinded study. Please answer the questions to the best of your ability related to the dog you have been assigned for the duration of the spring semester based on recollection of your initial interactions with this dog at the beginning of the semester. If a behavior is not observed then please do not answer the question – such as aggression or vocalization.

1. The anxiety level that the dog that you have been working with this week has been:

A Lot worse   A little worse   no change   A little better   A lot better

2. The lethargy and somnolence that the dog you have been working with this week has been:

A Lot worse   A little worse   no change   A little better   A lot better

3. The ease and ability to work with this dog you have been working with this week has been:

A Lot worse   A little worse   no change   A little better   A lot better

4. The behavior of biting, nipping or mouthing with this dog you have been working with this week has been:

A Lot worse   A little worse   no change   A little better   A lot better

5. The behavior of jumping up on you with this dog you have been working with this week has been

A Lot worse   A little worse   no change   A little better   A lot better

6. Vocalization in the form of barking and whining behavior with the dog you have been working with this week has been:

A Lot worse   A little worse   no change   A little better   A lot better

Supplemental Table S1. LC-MS/MS parameters for cannabinoid detection.

Compound name	Precursor Ion	Product Ions	Polarity
11-NOR-COOH-Δ9-THC-GLUCURONIDE	519.56	343.1/299	Negative
11-OH-Δ9-THC	331.47	313.8/193.3/122.9	Positive
6-OH-CBD	329.45	311.1/173.1/157.8	Negative
7-COOH-CBD	343.43	299.1/297/231	Negative
7-OH-CBD	329.45	311.1/261.1/178.9	Negative
CBC	315.47	193.4/123.1/93.1	Positive
CBCA	357.46	339.1/313.1/191	Negative
CBD	315.47	193.4/123.3/93	Positive
CBDA	359.48	341.3/261.5/219.4	Positive
CBGA	361.5	219.4/149.1/135.1	Positive
D3-11-OH-Δ9-THC	334.49	316.4/196.3/105.3	Positive
D3-7-OH-CBD	332.7	172.9/314.2/264.1	Negative
D3-CBD	318.49	196.3/123.2/93.1	Positive
D3-Δ9-THC	318.49	196.4/123.2/93.0	Positive
D3-THCA	360.49	316.1/248.0/194.0	Negative
D9-THC	315.47	193.4/123.1/93.0	Positive
THCA	357.46	313.1/245/191	Negative

Product ions consist of first value as quantified ion and additional ions were utilized as qualifier ions.

Supplemental Table S2: Percent Accuracy and percent coefficient of variation of assay. Analysis was done in triplicate on three days.

Compound	Accuracy (%)	%CV
7-COOH-CBD	102.0	15.5
11-NOR-COOH-Δ9-THC-GLUCURONIDE	115.5	8.8
6-OH-CBD	109.6	8.9
7-OH-CBD	96.4	16.3
11-OH-Δ9-THC	91.6	13.7
CBDA	90.6	12.7
CBGA	117.2	17.1
CBD	89.7	12.1
Δ9-THC	103.3	4.2
CBC	110.7	11.1
THCA	98.0	3.2
CBCA	115.6	10.2
